# The N-terminal length and side-chain composition of CXCL13 affect crystallization, structure and functional activity

**DOI:** 10.1107/S2059798320011687

**Published:** 2020-09-25

**Authors:** Eric M. Rosenberg, James Herrington, Deepa Rajasekaran, James W. Murphy, Georgios Pantouris, Elias J. Lolis

**Affiliations:** a Department of Pharmacology, Yale School of Medicine, New Haven, CT 06520, USA; b Yale Center for Molecular Discovery, Yale West Campus, West Haven, CT 06516, USA

**Keywords:** chemokines, G protein-coupled receptors, CXCL13, CXCR5, agonists

## Abstract

CXCL13 is the cognate chemokine agonist of CXCR5, a class A G-protein-coupled receptor (GPCR) that is essential for proper humoral immune responses. Using a ‘methionine scanning’ mutagenesis method on the N-terminus of CXCL13, which is the chemokine signaling region, it was shown that minor length alterations and side-chain substitutions still result in CXCR5 activation. This observation indicates that the orthosteric pocket of CXCR5 can tolerate these changes without severely affecting the activity. The introduction of bulk on the ligand was well tolerated by the receptor, whereas a loss of contacts was less tolerated. Furthermore, two crystal structures of CXCL13 mutants were solved, both of which represent the first uncomplexed structures of the human protein. These structures were stabilized by unique interactions formed by the N-termini of the ligands, indicating that CXCL13 exhibits substantial N-terminal flexibility while the chemokine core domain remains largely unchanged. Additionally, it was observed that CXCL13 harbors a large degree of flexibility in the C-terminal extension of the ligand. Comparisons with other published structures of human and murine CXCL13 validate the relative rigidity of the core domain as well as the N- and C-terminal mobilities. Collectively, these mutants and their structures provide the field with additional insights into how CXCL13 interacts with CXCR5.

## Abstract 2

The chemokine CXCL13 relies on its N-terminus for signaling, but minor length and side-chain variations still result in a agonistic forms of the protein. Two crystal structures reveal that both the N-terminus and the C-terminal extension of the protein are highly flexible, whereas the core domain of the protein is rigid.

## 1. Introduction   

Chemokines and their cognate G-protein-coupled receptors (GPCRs) are critical for proper immune surveillance, inflammation, wound repair and development. Chemokines are proteins of 8–12 kDa in size and are secreted after being expressed, where they are able to interact with their target receptors on leukocytes (Allen *et al.*, 2007 ▸). Chemokines function as agonists, and upon binding to their target GPCRs they induce receptor activation and subsequent signaling through G proteins in the Gα_i_ and Gα_q_ families (the former for most chemokine receptors), which lead to the inhibition of adenylyl cyclases and the mobilization of intracellular Ca^2+^ stores, respectively (Verkaar *et al.*, 2014 ▸; Zhang & Shi, 2016 ▸). These signaling events are integrated to ultimately cause the chemotaxis (*i.e.* directed cell migration) of immune cells in a concentration-dependent manner towards the highest concentration of ligand, thereby allowing chemokines to regulate the biological processes mentioned above. Despite highly variable sequence homologies (ranging from approximately 20% to 90%), all chemokines exhibit very similar tertiary structures (Allen *et al.*, 2007 ▸). As elucidated from structural data on many chemokines, this conserved tertiary structure consists of a disordered N-terminal region (6–10 amino acids, referred to here as the chemokine N-terminus), followed by a long loop known as the ‘N-loop’ ending in a short 3_10_-helix, a three-stranded β-sheet and a C-terminal α-helix. Two or four conserved cysteines (which classify the chemokine ligands into four families) form disulfide bonds that stabilize the overall topology (Allen *et al.*, 2007 ▸; Fernandez & Lolis, 2002 ▸). Importantly, it is known that the N-termini of chemokines are key mediators of agonistic activity, and that the deletion or modification of the N-terminus frequently results in variants that bind cognate receptors with relatively high affinity to function as antag­onists or, instead, suffer significant losses in binding affinity (Fernandez & Lolis, 2002 ▸; Allen *et al.*, 2007 ▸; Chevigné *et al.*, 2011 ▸).

CXCR5 [chemokine (C-*X*-C motif) receptor 5] is a class A GPCR that binds to a single ligand, CXCL13 [chemokine (C-*X*-C motif) ligand 13] (Tan *et al.*, 2018 ▸). CXCL13 is an agonist of CXCR5, activating the receptor, resulting in downstream calcium signaling and ultimately the chemotactic response for which chemokines are named (Gunn *et al.*, 1998 ▸; Legler *et al.*, 1998 ▸). CXCR5 is expressed on B cells as well as subsets of T cells known as T follicular helper (TFH) cells and is required for proper B-cell homing to discrete compartments within the tissues where CXCL13 is expressed (Hussain *et al.*, 2019 ▸; Moser, 2015 ▸; Förster *et al.*, 1996 ▸). In particular, CXCR5 is expressed in secondary lymphoid organs such as the spleen, lymph nodes and Peyer’s patches (Tan *et al.*, 2018 ▸; Hussain *et al.*, 2019 ▸), where it functions to keep B cells clustered into follicles such that interaction with TFH cells is easily accomplished to induce the formation of germinal centers (Moser, 2015 ▸). Germinal center activity leads to long-term immunity by mediating affinity maturation of antibodies to yield those that exhibit high affinities against target antigens, as well as the development of antibody-secreting plasma cells and memory B cells (Moser, 2015 ▸; Denton *et al.*, 2019 ▸).

CXCL13 has been less well studied than other chemokines. To date, there have been no studies aimed at understanding the significance of the N-terminus of CXCL13 and how the perturbation of its primary structure affects its ability to activate CXCR5. We sought to study how simple N-terminal modifications of CXCL13 would affect its activity. In particular, we utilized a ‘methionine scanning’ technique to both lengthen and shorten the N-terminus of CXCL13 by one residue, as well as to perform side-chain substitutions on its first amino acids. Our mutants resulted in agonistic variants of varying potency and efficacy despite these changes, demonstrating that CXCR5 is able to accommodate multiple different CXCL13 N-terminal sequences to trigger a functional effect. Introduction of bulk into the orthosteric cavity was better tolerated than loss of contacts. Furthermore, we were able to utilize two of these mutants to solve the first uncomplexed crystal structures of human CXCL13. These structures allowed us to observe that the core domain of CXCL13 (*i.e.* everything except its N- and C-termini) is fairly rigid, whereas its N-terminus exhibits striking flexibility as well as a propensity to form both intramolecular and intermolecular β-strand interactions. Additionally, we observed that the C-terminal extension (*i.e.* the residues after the C-terminal α-helix) of CXCL13 is incredibly dynamic, with multiple trajectories away from the core domain. Comparisons of two other published structures of human CXCL13 bound to single-chain variable fragment (scFv) molecules (Tu *et al.*, 2016 ▸) as well as two solution structures of murine CXCL13 (Monneau *et al.*, 2017 ▸) confirmed our finding of the rigidity of the core domain as well as the mobilities of the N- and C-termini. Collectively, our results provide the scientific community with a case study as to how some chemokine receptors can tolerate minor length and side-chain variation in the N-termini of their ligands and retain activity, as well as providing two unique structures of human CXCL13.

## 2. Materials and methods   

### 2.1. Cloning of CXCL13 constructs   

The human CXCL13 gene (residues 23–109, which represent the mature protein sequence without the secretion signal) was codon-optimized for expression in *Escherichia coli* and was subsequently purchased from GenScript in the pUC57 plasmid. The gene was subsequently digested with the restriction endonucleases NdeI and BamHI (New England Biolabs) and ligated into the pET-22b plasmid (Novagen), cut with the same enzymes, via T4 DNA ligase (New England Biolabs). The ligated product was transformed into XL10-Gold Ultracompetent cells (Agilent) and plated onto LB–ampicillin plates overnight to select for clones with the insert. Individual clones were grown in LB–ampicillin medium and were miniprepped with commercial miniprep kits (Qiagen). Sequencing of purified plasmid DNA from individual clones with the standard T7 reverse primer confirmed the insertion of the gene. The final construct contained an open reading frame (ORF) with human CXCL13 alone, without any tags for purification, but with an N-terminal initiating methionine (ultimately producing Met CXCL13).

The generation of N-terminal mutants was achieved via mutagenic oligonucleotides (Supplementary Table S1) using Met CXCL13 in the pET-22b construct described above as a template. The mutagenesis procedure was performed via site-directed mutagenesis using *PfuTurbo* polymerase (Agilent) followed by subsequent digestion of the methylated template plasmid DNA strands with DpnI (New England Biolabs) for 1–2 h at 37°C. The mutagenized DNA was transformed into XL10 cells and selected as described above, and individual clones were sequenced with T7 reverse primer. Each mutation retained the initiating methionine prior to the ORF encoding the designed mutations.

To produce wild-type CXCL13 (*i.e.* lacking the initiating methionine), site-directed mutagenesis was performed on Met CXCL13 in pET-22b as described above with primers designed to insert an enterokinase cleavage (sequence DDDDK) site between the initiating methionine and the first residue of the mature CXCL13 sequence (Supplementary Table S1). Miniprepped DNA from individual clones was sent out for sequencing with the T7 forward and reverse primers to confirm the insertion of the cleavage site. This construct was referred to as ‘EK-CXCL13’, but ultimately produced wild-type CXCL13 after removal of the N-terminal methionine and enterokinase cleavage site during purification.

### 2.2. Purification of recombinant CXCL13 constructs   

All CXCL13 constructs cloned into pET-22b were transformed into *E. coli* BL21(DE3) cells (New England Biolabs) and plated onto LB–ampicillin plates. Individual colonies were picked and tested for their ability to express the CXCL13 constructs upon the administration of isopropyl β-d-1-thio­galactopyranoside (IPTG; 1 m*M* final concentration). The colonies with the greatest expression were used to make a 20% glycerol stock and were used for subsequent purifications.

To produce CXCL13 constructs without an enterokinase cleavage step, LB–ampicillin medium was inoculated (1:100) with *E. coli* BL21(DE3) cells containing the CXCL13 construct of choice. They were grown at 37°C, were allowed to reach an OD_600_ of 0.6–0.8 and were then treated with IPTG (1 m*M* final concentration) to initiate induction. The cells were allowed to produce CXCL13 for 3–4 h at 37°C following induction. The cell pellets were collected by centrifugation and frozen at −20°C for later use. The thawed cells were resuspended in 20 m*M* Tris, 20 m*M* NaCl pH 7.4 (40 ml per litre of initial culture medium) and treated with three tablets of cOmplete Mini EDTA-free protease-inhibitor cocktail (Roche Life Science). Cell clumps were broken up and subsequently sonicated on ice to lyse the cells. The cell lysate was centrifuged for 25 min at 16 000 rev min^−1^ (31 000*g*) in a Beckman Coulter JA-20 rotor prechilled to 4°C and the supernatant was discarded. Cell pellets were resuspended in wash buffer *A* (100 m*M* Tris pH 8, 5 m*M* EDTA, 5 m*M* DTT, 2 *M* urea, 2% Triton X-100; 40 ml per litre of initial culture medium), fully homogenized via Dounce homogenization and centrifuged again with the same parameters as listed above. The wash was performed twice more (three washes with wash buffer *A*) and a fourth wash was performed with wash buffer *B* (100 m*M* Tris pH 8, 5 m*M* EDTA, 5 m*M* DTT). Purified inclusion bodies were then solubilized in solubilization buffer (100 m*M* Tris, 6 *M* guanidine hydrochloride pH 8; 20 ml per litre of initial culture medium) and centrifuged as before. The supernatant was collected and then added dropwise to refolding buffer (100 m*M* Tris pH 8, 5 m*M* EDTA, 0.2 m*M* oxidized glutathione, 1 m*M* reduced glutathione; 100 times the volume of solubilized protein) prechilled to 4°C. Refolding was allowed to continue at 4°C overnight (∼18 h).

Refolded protein was filtered using Whatman filter paper and a Buchner funnel, the pH was lowered to ∼7.1 by the addition of hydrochloric acid and a final filtration step was performed using a 0.45 µm filter in order to remove precipitant. The protein was loaded onto a 5 ml HiTrap SP FF column (a cation exchanger; GE Healthcare Life Sciences) pre-equilibrated with running buffer (10 m*M* potassium phosphate buffer, 5 m*M* EDTA pH 7), washed with ten column volumes (CVs) of running buffer and then eluted with a 20 CV linear gradient to elution buffer (10 m*M* potassium phosphate buffer, 5 m*M* EDTA, 1 *M* NaCl pH 7). Eluates were analyzed via sodium dodecyl sulfate–polyacrylamide gel electrophoresis (SDS–PAGE) and fractions containing CXCL13 were pooled, filtered with a 0.45 µm filter and frozen at −20°C for later use. Thawed protein was then further purified via HPLC with POROS 20 R2 reversed-phase resin (Thermo Fisher) using a 20–40% gradient of acetonitrile in H_2_O (both mobile phases contained 0.1% trifluoroacetic acid). CXCL13 eluted at roughly 30% acetonitrile, and the fractions were subsequently diluted to ∼10% acetonitrile using distilled water. The protein was then concentrated at 4°C using Amicon Ultra-15 Centrifugal Filter Units with a 3000 molecular-weight cutoff (EMD Millipore) and washed several times with cold 10% aceto­nitrile to remove excess trifluoroacetic acid. The final concentrate (∼2–4 ml) was flash-frozen in liquid nitrogen and lyophilized overnight, and purified CXCL13 was resuspended in sterile distilled water. The concentration of the protein was quantified with a NanoDrop system (Thermo Scientific) using theoretical protein sizes and extinction coefficients (a value of 14 180 *M* ^−1^ cm^−1^ was used for the latter for all constructs), and the purified protein was stored at 4°C until needed. The final yield was typically ∼3–4 mg per litre of initial culture medium.

To produce wild-type CXCL13 from the EK-CXCL13 construct (Supplementary Table S1), an enterokinase cleavage step was added to the above protocol between cation exchange and reversed-phase HPLC. Specifically, the fractions from cation exchange containing CXCL13 were first buffer-exchanged into enterokinase cleavage buffer (20 m*M* Tris pH 7.4, 50 m*M* NaCl, 2 m*M* CaCl_2_) using Amicon Ultra-15 Centrifugal Filter Units with a 3000 molecular-weight cutoff (EMD Millipore). EK-CXCL13 was then cleaved using recombinant enterokinase (Novagen) at a ratio of 100 µg CXCL13 to one unit of recombinant enterokinase (diluted in the provided storage buffer to one unit per microlitre). To cleave 100 µg of protein, a 350 µl total volume (including enterokinase) was used. The proteolysis was allowed to proceed overnight (∼16–18 h), after which the reaction was filtered through a 0.45 µm filter and promptly loaded onto the reversed-phase HPLC column to separate wild-type CXCL13 and enterokinase. All other subsequent steps were the same as described above.

### 2.3. Calcium-flux assays   

Calcium-flux assays were conducted at the Yale Center for Molecular Discovery (YCMD) using a FLIPR imaging system (Molecular Devices). For each assay, division-arrested HEK-293T cells expressing CXCR5 with an N-terminal FLAG tag (protein sequence DYKDDDDK; Multispan) were thawed and utilized on the same day. Specifically, one vial of the cells (containing 4 × 10^6^ cells) was removed from storage in liquid nitrogen and was heated to 37°C, after which the cells were diluted into 5 ml Dulbecco’s Modified Eagle Medium (DMEM) containing 10% FBS and penicillin/streptomycin and then centrifuged to form a pellet. The supernatant was removed and the cells were resuspended in 3 ml Hank’s Balanced Salt Solution (HBSS) containing calcium and magnesium (Gibco) and then counted. The cell count was adjusted to 3 × 10^5^ cells ml^−1^ with HBSS, and 30 µl of the cell solution (9000 cells) was then plated into each well of a 384-well plate coated with poly-d-lysine (Greiner). The cells were allowed to settle for 10 min, and were then centrifuged for 5 min at 200*g*. A no-wash calcium dye (BD Bio­sciences) was prepared according to the manufacturer’s protocol, after which 10 µl was added to each well of the 384-well plate. The cells with dye were then incubated for 30 min at 37°C followed by 30 min at room temperature, at which point they were ready for the immediate addition of test ligands and monitoring in the FLIPR system.

Test ligands were prepared by dilution into an HBSS/Ca^2+^/Mg^2+^ solution containing BSA such that, after addition to the cells, the BSA was at a final concentration of 0.1%. For each assay, a final concentration of 2 µ*M* ionomycin (Sigma) was used as a positive control to show the maximum calcium-flux response elicited via receptor-independent release of internal calcium stores (Morgan & Jacob, 1994 ▸), whereas HBSS/Ca^2+^/Mg^2+^ was used as a negative control to show no response. Each test sample was monitored with six replicates for each concentration, and the final volumes in the wells at the end of the assay were 66.6 µl per well. After collecting a baseline reading, the fluorescent calcium response was monitored every few seconds for several minutes, and the results for each point were plotted using *GraphPad Prism* as the maximum fluorescence response elicited.

### 2.4. Crystallization and X-ray data collection and processing   

Both Met CXCL13 and Δ1L2M CXCL13 were diluted to 12 mg ml^−1^ in distilled water and were subsequently used to set up crystal trays. A Mosquito system (TTP Labtech) was used to screen for crystals using several crystal screens from Hampton Research as well as the Wizard kit from Emerald BioSystems by the hanging-drop method. For Met CXCL13, crystals were found to form in formulation No. 19 of the Hampton Research PEGRx 2 kit [0.1 *M* NaCl, 0.1 *M* bis-tris propane, 25%(*w*/*v*) polyethylene glycol (PEG) 1500 pH 9] using a 1:1 ratio of protein to precipitant (400 nl each), and this formulation was used to set up a 96-well tray of crystals. Crystals grew in these conditions at 20°C over the course of a few days. Similarly, Δ1L2M CXCL13 was able to form crystals in formulation No. 16 of the Hampton Research PEGRx 1 kit [0.1 *M* citric acid pH 3.5, 14%(*w*/*v*) PEG 1000] using a 1:1 ratio of protein to precipitant (400 nl each). A crystal of Δ1L2M CXCL13 was harvested after approximately one month and was used directly from the screening tray.

CXCL13 crystals were dipped into the appropriate mother liquor as a cryoprotectant, and were mounted onto an in-house rotating copper-anode X-ray generator (wavelength 1.54 Å) and data-collection system. A cryostream set to a temperature of 100 K was used to stabilize the crystals, and full data sets were collected to 1.88 or 2.52 Å resolution for Met and Δ1L2M CXCL13, respectively, using a Dectris PILATUS detector (see Table 1 ▸ for details of data collection). The data sets were processed using *HKL*-2000 and the intensity data were then imported into the *CCP*4 suite (Winn *et al.*, 2011 ▸; Met CXCL13) or *Phenix* (Liebschner *et al.*, 2019 ▸; Δ1L2M CXCL13) as an .mtz file. The structure of Met CXCL13 was solved first; to solve the phase problem, a monomer of CXCL10 was generated from PDB entry 1o7y (Swaminathan *et al.*, 2003 ▸) by deleting all atoms other than those in chain *A*, and this new coordinate file was used to perform molecular replacement with *Phaser* (McCoy *et al.*, 2007 ▸) in *CCP*4. The output .mtz file was used by *Buccaneer* (Cowtan, 2006 ▸) in *CCP*4 to autobuild CXCL13, and the initial model was iteratively refined via *REFMAC*5 (Murshudov *et al.*, 2011 ▸) and manual refinement in *Coot* (Emsley *et al.*, 2010 ▸) (see Table 1 ▸ for details of the refinement). An iterative-build composite OMIT map was generated in *Phenix* in order to ensure that the model did not contain phase bias from our molecular-replacement model. The refined coordinates were originally deposited in the PDB as entry 4zai, but were subsequently superseded by entry 7jny after rescaling in *HKL*-2000 and re-refinement using *Phenix* in order to increase the resolution of the structure. The final structure exhibited no Ramachandran outliers and 0% of the amino acids were in the allowed region.

The structure of Δ1L2M CXCL13 was solved using the structural coordinates of Met CXCL13 for molecular replace­ment. Specifically, the N-terminal residues of Met CXCL13 up to the C-*X*-C motif (residues 11–13 of the mature protein sequence, with the initiating methionine defined as position 0) were removed and this new coordinate file was used to perform molecular replacement with *Phaser* in *Phenix*. The output structural coordinates were then examined in *Coot*, and the N-terminus was manually built using baton mode for each of the seven monomers in the asymmetric unit. The coordinates containing the N-terminus were then iteratively refined in *Phenix* and in *Coot* (see Table 1 ▸ for details of the refinement). Phase bias was avoided by the examination of an iterative-build composite OMIT map generated in *Phenix*. The final structure exhibited no Ramachandran outliers and ∼2% of residues were in the allowed region.

To visualize the structures, we utilized *PyMOL* (Schrödinger). Two publicly available *PyMOL* scripts were utilized to prepare some of the images, namely color_h and anglebetweenhelices. All images of the proteins shown in the figures were created using the png command in *PyMOL* with ray-tracing turned on.

### 2.5. R.m.s.d. calculations   

To calculate the r.m.s.d. values between various CXCL13 monomers, the structures were first visualized in *PyMOL* and residues to be excluded from the analyses were manually deleted. The truncated CXCL13 monomers were then saved as individual PDB files and were subsequently uploaded to the *SuperPose* web server (Maiti *et al.*, 2004 ▸) to perform r.m.s.d. calculations. R.m.s.d. values for both C^α^ atoms alone and all backbone atoms were manually recorded into the relevant tables.

## 3. Results and discussion   

### 3.1. Initial characterization of Met CXCL13 as an N-terminal extension mutant   

Chemokines are easily expressed in large quantities in *E. coli* for purposes of structural determination using X-ray crystallography or NMR, as well as for conducting both biophysical and biological studies. Using *E. coli*, chemokines tend to retain the initiating methionine at position 0 (Met0) that is used to initiate the transcription of the mature protein sequence, but mammalian expression instead utilizes secretion sequences that, after cleavage, result only in the physiologically mature wild-type (WT) sequence beginning at position 1. In *E. coli*, retention of the N-terminal Met0 can be attributed to differences in the enzymatic efficiency of methionine aminopeptidase (Hirel *et al.*, 1989 ▸; Xiao *et al.*, 2010 ▸). Chemokines such as CXCL12 [chemokine (C-*X*-C motif) ligand 12] and CCL5 [chemokine (C-C motif) ligand 5, also known as RANTES] retain an initiating methionine after purification (Murphy *et al.*, 2007 ▸; Proudfoot *et al.*, 1996 ▸). The function can vary significantly among chemokines with the initiating methionine present. While CXCL12 functions as an agonist with the initiating methionine present (Rosenberg *et al.*, 2019 ▸), CCL5 instead functions as a receptor antagonist (Proudfoot *et al.*, 1996 ▸), indicating that some chemokine receptors can accommodate an additional residue at position 0 to trigger activity, whereas others cannot. We wished to observe whether this was the case with CXCR5, and thus we purified both recombinant WT CXCL13 as well as Met CXCL13 from *E. coli* (Table 2 ▸). To obviate the issue of retaining the initiating methionine with WT CXCL13, we mutagenized the Met CXCL13 construct to introduce an enterokinase cleavage site (residues DDDDK) between the initiating methionine and the native N-terminus of CXCL13 (the EK-CXCL13 construct; Supplementary Table S1). The purification of this construct was performed in a similar manner as the other CXCL13 constructs described in this paper, with an additional entero­kinase cleavage step performed to remove the MDDDDK sequence, yielding the WT protein. Both WT and Met CXCL13 were verified to be the correct sizes via intact liquid-chromatography mass spectrometry (LC-MS, performed at the Mass Spectrometry and Proteomics Resource at Yale’s Keck Biotechnology Resource Laboratory), although we did observe that a small fraction of Met CXCL13 had lost the initiating methionine, resulting in the production of WT CXCL13 (Supplementary Fig. S1).

**Figure 1:**
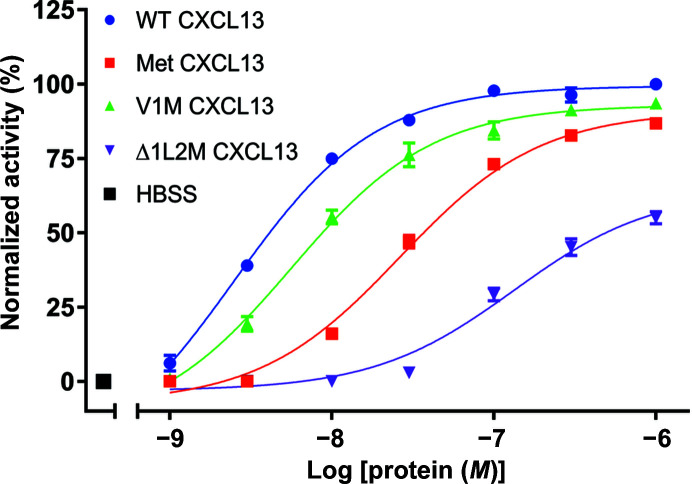
Calcium-flux assays using CXCL13 constructs. For all curves, error bars represent the standard error of the mean (SEM) derived from three independent experiments. The CXCL13 constructs described in Table 2 ▸ were administered onto HEK-293T cells expressing human CXCR5, and the maximum calcium response was used to generate the concentration–response curves.

To serve as a functional assay to determine the CXCR5-dependent activities of our CXCL13 constructs, we performed calcium-flux experiments with cells expressing human CXCR5 (Fig. 1 ▸). Upon administering both WT and Met CXCL13 to these cells in a dose-dependent manner, we observed that both functioned as agonists, albeit with differing potencies and efficacies. In particular, we found that WT CXCL13 had an observed half-maximal effective concentration (EC_50_) value of 2.49 n*M*, whereas Met CXCL13 had an EC_50_ of 26.3 n*M* (Fig. 1 ▸, Table 2 ▸). In terms of efficacy, we defined the maximum activity elicited by WT CXCL13 acting on CXCR5 at the highest concentration tested (1 µ*M*) to be 100% (defining it as a full agonist) and our negative control of buffer alone to equate to 0% activity; our receptor-independent positive control, 2 µ*M* ionomycin, produced a response of ∼119.6% relative to WT CXCL13 (data not shown). Using these parameters, we observed that Met CXCL13 had a maximum efficacy of 86.8% when administered at 1 µ*M*, classifying it as a partial agonist of CXCR5 (Fig. 1 ▸, Table 2 ▸). These results indicate that CXCR5 can accommodate an extra amino acid on the N-terminus of CXCL13 for both ligand binding and receptor activation, but with some loss in potency and efficacy.

### 3.2. Characterization of additional N-terminal mutants of CXCL13   

After initially observing that Met CXCL13 functioned as an agonist of CXCR5, we then chose to generate two new N-terminal variants of CXCL13 using a ‘methionine scanning’ approach, in which we allowed the initiating methionine to replace Val1 of the WT protein, and a separate mutant in which Val1 was deleted and Leu2 was mutated to methionine (V1M and Δ1L2M mutants, respectively; Table 2 ▸). Albeit with different research efforts in mind, such a technique has been used in the past to study fragments of the yeast cytochrome *c* protein and their ability to re-ligate following cleavage with cyanogen bromide (Woods *et al.*, 1996 ▸). We reasoned that the V1M and Δ1L2M mutants would allow us to observe the degree to which CXCR5 could accommodate not only length changes but also side-chain variations in the N-terminus of CXCL13. Although methionine is the most well tolerated amino acid when performing substitutions (Gray *et al.*, 2017 ▸), we sought to utilize it in our mutants over the common choice of alanine as the former would allow us to introduce longer side chains into the orthosteric site of CXCR5 as opposed to removing them with alanine. Since Met CXCL13 was able to activate CXCR5 (Fig. 1 ▸), it seems that the orthosteric site of CXCR5 is capable of tolerating additional bulk compared with the WT CXCL13 N-terminus. The degree to which these longer side chains would be tolerated at positions 1 and 2 (the positions closest to the unnatural position 0 in Met CXCL13) was unclear, but we hypothesized that a longer methionine side chain would be accommodated. Moreover, keeping the N-terminal methionine constant would allow the easiest interpretation of the ligand activity since it allows comparison with Met CXCL13 by moving the methionine away from the orthosteric pocket of CXCR5. These constructs were purified from *E. coli* and their identities were verified via LC-MS (Supplementary Fig. S1).

We performed additional calcium-flux experiments with the V1M and Δ1L2M constructs for comparison with WT and Met CXCL13. We found that both V1M and Δ1L2M CXCL13 functioned as agonists, with EC_50_ values of 5.64 and 133 n*M*, respectively. In terms of efficacy, V1M CXCL13 was the most efficacious construct other than WT CXCL13, attaining 93.6% activity at 1 µ*M*, whereas Δ1L2M CXCL13 was only able to achieve a mere 55.1% activity at the same concentration (Fig. 1 ▸, Table 2 ▸). The results indicate that in addition to the Met CXCL13 insertion mutant, CXCR5 is also able to accommodate N-terminal CXCL13 mutants with side-chain variations and a shortened length. The most active constructs were WT and V1M CXCL13, suggesting that despite the V1M mutation, the length of CXCL13 is fine-tuned to elicit the highest degree of CXCR5 activation. Both insertions and deletions (Met and Δ1L2M, respectively) could still elicit receptor activation, although the loss of N-terminal (*i.e.* position 1) interactions between the Δ1L2M CXCL13 ligand and CXCR5 seems to reduce either the binding affinity of the ligand and/or its ability to trigger the CXCR5 conformational changes leading to activation. The L2M mutation may also contribute to the change in CXCR5 activation, although the hydrophobic nature of position 2 is retained. In the case of Met CXCL13, the interactions of WT CXCL13 are maintained for position 1, presumably contributing to ligand binding, but the presence of an additional methionine may cause steric clashes that affect the receptor activation potential.

### 3.3. Crystal structure of Met CXCL13   

**Figure 2:**
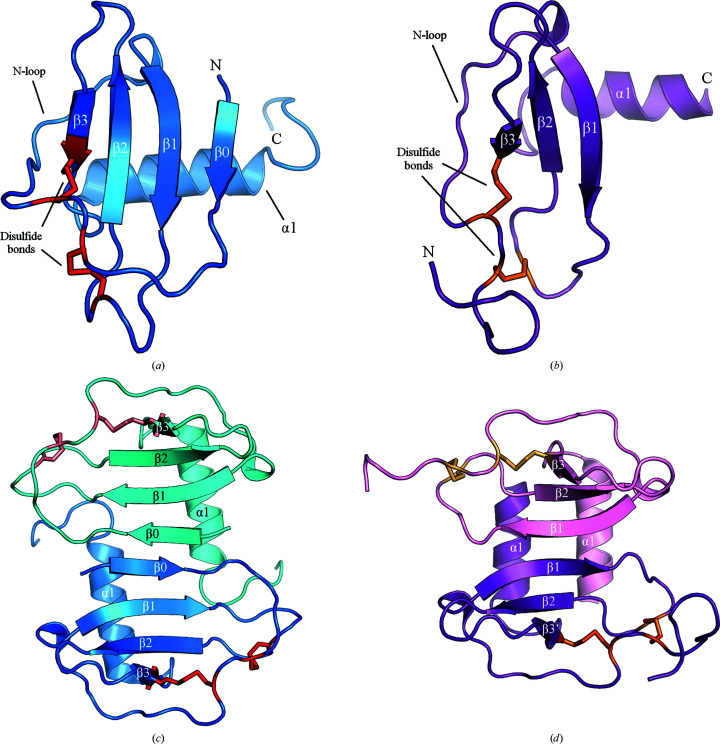
Met CXCL13 crystal structure compared with that of a canonical C-*X*-C chemokine. Data-collection and refinement statistics are shown in Table 1 ▸ and structural features are labeled. (*a*) Met CXCL13 crystallizes in space group *P*3_1_21 with one monomer in the asymmetric unit. Disulfide bonds are depicted in red. (*b*) As a representative example, a monomer of CXCL12 (from PDB entry 2nwg; Murphy *et al.*, 2007 ▸) is shown. Disulfide bonds are depicted in orange. (*c*) The Met CXCL13 structure contains one monomer in the asymmetric unit, but a symmetry mate shows that a dimer is formed between the unique β0 strands, allowing an overall eight-stranded unit to be seen. (*d*) CXCL12 is once again used as a comparison to demonstrate how C-*X*-C chemokines typically dimerize via the β1 strands in the core domain, leading to an overall six-stranded unit.

To put the functional data into a structural context, we next attempted to crystallize the CXCL13 variants that we had purified. Given that the structure of CXCR5 has also not been solved, we reasoned that any structural information about the ligand and receptor pair would be useful. We were able to solve the crystal structure of Met CXCL13 to 1.88 Å resolution. We utilized the structure of CXCL10 [chemokine (C-*X*-C) motif ligand 10] (Swaminathan *et al.*, 2003 ▸) to perform molecular replacement and generate an initial model that was then iteratively refined to generate the final structure. Upon solving the structure (PDB entry 7jny; Table 1 ▸), we observed that in addition to exhibiting a canonical chemokine core domain (consisting of an N-loop region followed by a three-stranded β-sheet and a C-terminal α-helix), Met CXCL13 also featured an N-terminus that formed an additional parallel β-strand interaction (β0) with the β1 strand, leading to a four-stranded β-sheet (Fig. 2 ▸ *a*). This is in contrast to almost all other chemokine structures, in which the N-terminus is typically flexible and disordered [see Met CXCL12 (PDB entry 2nwg; Murphy *et al.*, 2007 ▸) in Fig. 2 ▸(*b*)]. It is interesting to note that this feature has only been seen one other time: in the solution structure of murine CXCL13 (Monneau *et al.*, 2017 ▸). Further inspection of Met CXCL13 indicated that there is one monomer per asymmetric unit that forms a crystallographic dimer with a symmetry mate. The dimer is formed between the β0 strands in both monomers, leading to an overall eight-stranded β-sheet (Fig. 2 ▸ *c*). Once again, this is in contrast to typical C-*X*-C chemokines, which are known to form dimers between the β1 strands, leading to an overall six-stranded β-sheet (Allen *et al.*, 2007 ▸; Miller & Mayo, 2017 ▸; see CXCL12 as a representative example; Fig. 2 ▸ *d*). Specifically, the two extra β0 strands lead to a translation of the two α-helices away from each other that is atypical of a C-*X*-C dimer.

**Figure 3:**
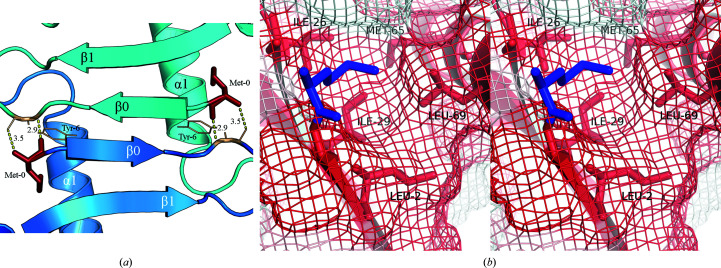
The initiating methionine (Met0) in the Met CXCL13 structure mediates both intermolecular and intramolecular interactions. Structural features are labeled. (*a*) The Met0 residues in the dimer seen in Fig. 2 ▸(*c*) (depicted in brick red) form intermolecular hydrogen bonds with Tyr6 (depicted in gold) in symmetry mates within the unit cell. Hydrogen-bond distances (in Å) are provided. (*b*) Stereoscopic image showing that the initiating methionine (depicted in blue) is stabilized by a hydrophobic groove in the core domain established by amino-acid residues Leu2, Ile26, Ile29, Met65 and Leu69 (depicted as red sticks). The hydrophobicity was determined using the normalized consensus hydrophobicity scale (Eisenberg *et al.*, 1984 ▸), in which red and white represent hydrophobic and hydrophilic amino acids, respectively. The surface of the protein (minus the initiating methionine) is shown as a mesh.

We next observed the specific interactions that allow the formation of the β0 strand in the Met CXCL13 structure. One set of interactions are the typical hydrogen bonds formed between the β0 and β1 strands to create the parallel β-strand interaction and the four-stranded β-sheet. We also observed that the initiating methionine itself (Met0) was responsible for helping to stabilize this unique feature with both intermolecular and intramolecular interactions. Specifically, Met0 allows intermolecular hydrogen bonds to be formed with Tyr6 in the symmetry mate that lead to the aforementioned crystallographic dimer (Figs. 2 ▸ *c* and 3 ▸ *a*). Additionally, we observed that the Met0 side chain occupies a hydrophobic pocket localized on the CXCL13 core domain formed by amino acids Leu2, Ile26, Ile29, Met65 and Leu69 (Fig. 3 ▸ *b*).

### 3.4. Crystal structure of Δ1L2M CXCL13   

**Figure 4:**
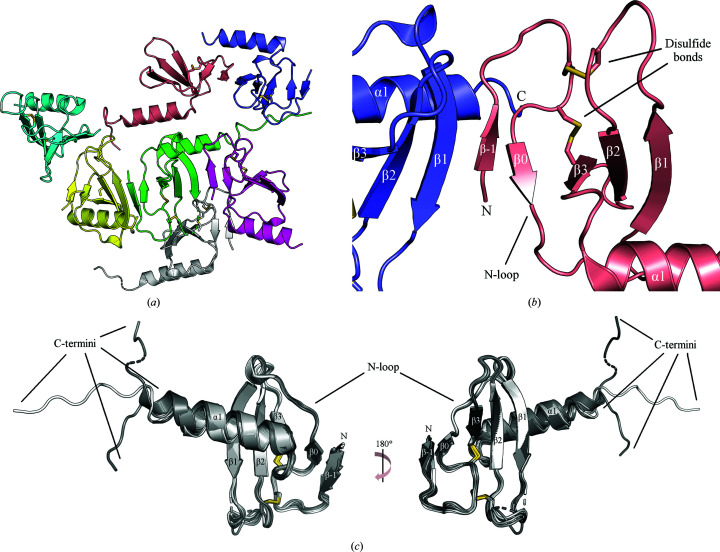
Δ1L2M CXCL13 crystal structure. Data-collection and refinement statistics are shown in Table 1 ▸ and structural features are labeled. Missing residues are shown as dashed lines. (*a*) Δ1L2M CXCL13 crystallizes in space group *P*12_1_1 with seven monomers in the asymmetric unit. Each monomer is colored a separate color for clarity. (*b*) The N-termini of the Δ1L2M CXCL13 monomers mediate interactions that collectively lead to the formation of a five-stranded β-sheet between two monomers. Note that the N-terminus of one monomer is visible, while the labeled C-terminus is that from another monomer. Only two monomers are shown for simplicity. (*c*) Alignments of all seven monomers in the asymmetric unit demonstrate that the N-terminus and core domain only exhibit minor alterations (see Supplementary Fig. S2 for calculated r.m.s.d. values).

We were able to solve a second crystal structure of a CXCL13 variant, Δ1L2M, to 2.52 Å resolution (PDB entry 6vgj; Table 1 ▸). This structure was solved by utilizing the existing Met CXCL13 core domain for molecular replacement and then manually building the N-terminus. While the Met CXCL13 structure exhibited one monomer in the asymmetric unit, we observed seven monomers of Δ1L2M CXCL13 in the asymmetric unit (Fig. 4 ▸ *a*). We were also very intrigued to note that once again the N-terminus of Δ1L2M CXCL13 formed additional β-strand interactions, although they were different from those seen in the Met CXCL13 structure. Specifically, we observed that the N-terminus of each monomer exhibits an extreme turn away from its core domain and then folds into a β-strand (β−1) which interacts in an antiparallel fashion with both the three-stranded core β-sheet from an adjacent monomer as well as an induced β-strand formed by the N-loop within the same monomer (β0). Ultimately, this results in the formation of an overall five-stranded β-sheet between the two monomers comprised of a three-stranded β-sheet of the core domain from one monomer and β0 and β−1 of the other (Fig. 4 ▸ *b*). Although this CXCL13 construct is a mutant and the oligomeric structure differs from other chemokines, we cannot ignore other chemokines that crystallize as larger aggregates. For example, CXCL12 also crystallizes as a decamer (Murphy *et al.*, 2010 ▸). We note that although this structure does highlight unique interactions between monomers, they look nothing like a canonical C-*X*-C chemokine dimer (Figs. 2 ▸ *d*, 4 ▸ *a* and 4 ▸ *b*).

By aligning the seven monomers found within the asymmetric unit of the Δ1L2M CXCL13 structure, we were able to observe that they are all extremely similar prior to their C-terminal ends, with only minor fluctuations found in their N-termini and in the β1–β2 loop region. Indeed, two monomers were observed to be lacking density corresponding to the residues found in the β1–β2 loop, highlighting the flexibility of this loop region (Fig. 4 ▸ *c*). All monomers exhibited density beginning at the N-terminal residue Met2, with the exception of one chain that began at Glu3. The final C-terminal residue varied between monomers, with the shortest monomer ending at Arg72 and the longest monomer ending at Lys83. By calculating the root-mean-square deviation (r.m.s.d.) of backbone atoms from each monomer beginning from residue Met2 (or Glu3 for one chain) up to Arg72, we observed that the values for any given combination of monomers ranged from 0.42 to 0.99 Å, indicating that the monomers were quite similar prior to their C-termini (Supplementary Fig. S2).

The alignments also showed that the largest observable differences between the monomers was in their C-terminal regions following the α1 helix, also known as the C-terminal extension. As mentioned above, while one monomer ended at Arg72, the longest monomer had visible density up to Lys83, only four residues short of the final residue found in mature CXCL13: Pro87. The next longest chains exhibited density up to Val81 and Pro80, respectively (the former did not have density that clearly corresponded to Leu77, and so it was not modeled into the structure). The coordinates of the C-terminal amino acids in these three longest monomers demonstrated that the C-terminal extension of CXCL13 is highly mobile, a phenomenon that we have been able to capture in a static crystal structure by simple alignments of its constituent monomers (Fig. 4 ▸ *c*). Such distinctive positions of the C-terminal extension, while indicative of extensive mobility in solution, were stabilized via crystal contacts and do not necessarily reflect genuine positions that would frequently occur in solution.

### 3.5. Comparisons of the Met and Δ1L2M CXCL13 crystal structures   

**Figure 5:**
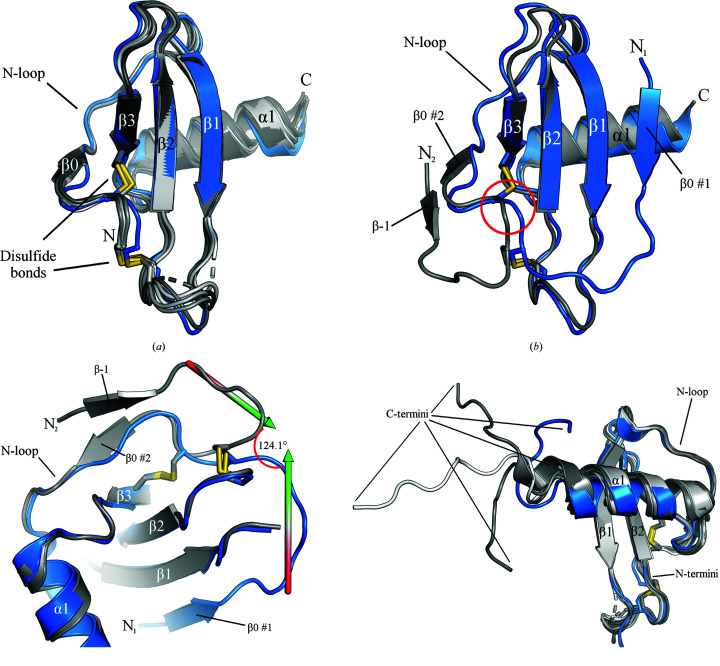
Comparisons between the Met and Δ1L2M CXCL13 crystal structures. Structural features are labeled. Missing residues are shown as dashed lines. (*a*) The seven core domains from the Δ1L2M CXCL13 structure (shades of gray) as well as that from the Met CXCL13 structure (blue) were overlaid to demonstrate their rigidity (see Supplementary Fig. S3 for calculated r.m.s.d. values). Note that the β0 strands localized in the N-loop only occur in the Δ1L2M CXCL13 monomers. (*b*) The Met CXCL13 monomer was overlaid with a single Δ1L2M CXCL13 monomer (chain *E*) to demonstrate the differences in the positions of their N-termini. N_1_ and β0 #1 in the figure refer to structural features in Met CXCL13, while N_2_, β0 #2 and β−1 in the figure refer to structural features in Δ1L2M CXCL13. The red circle indicates the branch point of the N-termini, which occurs close to the C-*X*-C motif. (*c*) The same monomers as shown in (*b*) are used to demonstrate that the angle between the main-chain atoms upstream of the C-*X*-C motif in the two structures was approximately 124.1°. Note that the β1–β2 loop is hidden for clarity. (*d*) Alignment of all seven Δ1L2M monomers and the Met CXCL13 monomer (minus their N-termini, which are hidden) emphasizes that the C-terminal extension of CXCL13 is incredibly flexible (see also Fig. 4 ▸ *c*).

To compare the similarities between the Met and Δ1L2M CXCL13 core domains, we first calculated the overall r.m.s.d. between each of the core domains (*i.e.* beginning at the C-*X*-C motif and ending at position Arg72 in the WT CXCL13 sequence) from the eight total monomers obtained from both structures. The r.m.s.d. values calculated using backbone atoms between any of the given combinations ranged from 0.32 to 1.07 Å, with the largest visible differences being seen in the β1–β2 and β2–β3 loops, in the N-loop and at the C-terminal end of the α1 helix (Fig. 5 ▸ *a* and Supplementary Fig. S3). These observations suggest that the core domain of CXCL13 is relatively static except for minor flexibility in loop regions and in the localization of the C-terminal α-helix.

To highlight the differences between the N-termini in our two CXCL13 structures, we aligned the Met CXCL13 structure with one of the seven monomers (chain *E*) from the Δ1L2M CXCL13 structure. As stated above, the core domains are relatively similar to one another, but there are striking differences in their N-termini prior to Cys13, the second cysteine located in the C-*X*-C motif. While the Met CXCL13 structure exhibits an N-terminus that wraps back up to form a β0 strand with β1 in the same monomer, the Δ1L2M CXCL13 structure has an induced β0 strand in its N-loop that interacts with the β−1 strand formed by the N-terminus in the same monomer (Fig. 5 ▸ *b*). We calculated the angle between main-chain atoms upstream of the branch point (Fig. 5 ▸ *b*, red circle) to be approximately 124.1°, once again emphasizing the immense flexibility of the N-terminus of the protein (Fig. 5 ▸ *c*). The resulting difference between the N-termini is approximately 26 Å when comparing the distances between the N atom of Met0 versus the N atom of Met2 in the Met and Δ1L2M CXCL13 structures, respectively. Similarly, we calculated a distance of approximately 24 Å when comparing the distance between the N atoms of Leu2 and Met2 in the Met and Δ1L2M CXCL13 structures, respectively (data not shown). Altogether, these measurements indicate that the flexibility of the CXCL13 N-terminus allows its initial amino acids to localize to relatively disparate positions in three-dimensional space.

Finally, we examined the alignments of all seven Δ1L2M CXCL13 monomers with that of the monomer from Met CXCL13 to see whether there were any differences in the C-terminal positions. Ending at Val79, we observed that the C-terminal extension from the Met CXCL13 structure had a trajectory that was different from any of those seen in the Δ1L2M CXCL13 structure, in that it coiled back towards the core domain as opposed to extending away from it as seen in the Δ1L2M monomers (Fig. 5 ▸ *d*), seemingly owing to steric hindrance with a symmetry mate. Yet again, while we note that the aligned monomers of CXCL13 from our structures allow us to capture the mobility of the C-terminal extension, we cannot necessarily interpret the positions in the crystal structures to match those that would be seen in solution, as the former were stabilized through crystal contacts.

### 3.6. Comparisons with WT CXCL13 in complex with scFv constructs   

**Figure 6:**
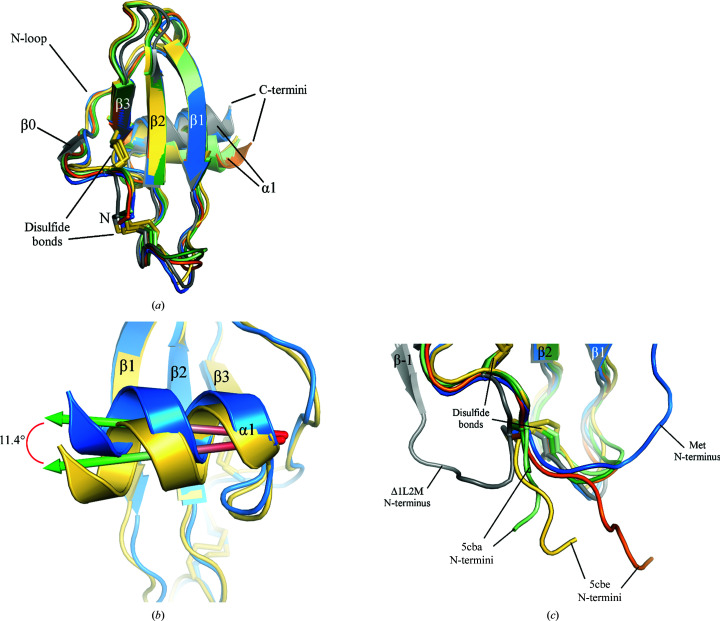
Comparisons between the Met and Δ1L2M CXCL13 crystal structures and the structures of CXCL13 found in PDB entries 5cba and 5cbe. Structural features are labeled. (*a*) The core domain from the Met CXCL13 structure (blue) and chain *E* of the Δ1L2M CXCL13 structure (gray) were overlaid with those from PDB entries 5cba (shades of green) and 5cbe (orange and yellow). The core domain exhibits relative rigidity between the structures (see Supplementary Fig. S4 for calculated r.m.s.d. values). Note that the β0 strand localized in the N-loop only occurs in the Δ1L2M CXCL13 monomer. (*b*) Close-up view of the change in the angling of the α1 helix in the structures with PDB codes 5cba and 5cbe. As a representative for each α1 helix position, the Met CXCL13 monomer was compared with one of the monomers (chain *F*) of PDB entry 5cbe. The angle between the two helical positions was found to be approximately 11.4°. (*c*) N-terminal trajectories between the structures of Met CXCL13, Δ1L2M CXCL13 and PDB entries 5cba and 5cbe. Note that only chain *E* of the Δ1L2M CXCL13 structure was used for clarity and that β−1 only occurs in this structure.

Two other structures of WT CXCL13 in complex with scFv constructs (PDB entries 5cba for scFv 3B4 and 5cbe for scFv E10) were published prior to this work (Tu *et al.*, 2016 ▸). In each of these structures two copies of the CXCL13–scFv complex exist within the asymmetric unit. To compare these structural coordinates of CXCL13 with our own, we aligned the four copies of CXCL13 obtained from PDB entries 5cba and 5cbe with the coordinates of both Met CXCL13 and chain *E* of Δ1L2M CXCL13 (we chose to utilize only a single chain of the Δ1L2M structure for simplicity). To compare the similarities of the core domains between the structures, we truncated the structures to begin with the C-*X*-C motif and to terminate at C-terminal position Val68, the latter being the last position with observed density in one of the monomers in PDB entry 5cbe (Fig. 6 ▸ *a*). Similar to the differences observed between the Met and Δ1L2M CXCL13 core domains (Fig. 5 ▸ *a*), the visible differences observed between the structures in this study and those found in the two CXCL13–scFv complexes are seen in the β1–β2 and β2–β3 loops, in the N-loop and in the α1 helix (Fig. 6 ▸ *a*). Indeed, the largest difference between our structures and those found in PDB entries 5cba and 5cbe were in the relative angles of the α1 helix with respect to the β-sheet (Figs. 6 ▸ *a* and 6 ▸ *b*). We calculated the angle between the two helical positions (using the coordinates of Met CXCL13 and chain *F* of PDB entry 5cbe) to be 11.4° (Fig. 6 ▸ *b*). This positioning of the α1 helix would indicate a major difference of the CXCL13 core domain, but closer examination of the structures with PDB codes 5cba and 5cbe demonstrates that the α1 helix is moved owing to interactions between scFv loop regions and symmetry mates within these structures (data not shown). Thus, this movement represents a conformational change owing to scFv binding and not intrinsic CXCL13 movements. To quantify the similarities of these core domains, we once again calculated r.m.s.d. values. The values calculated using backbone atoms ranged from 1.25 to 1.44 Å when comparing the Met CXCL13 core domain with those found in PDB entries 5cba and 5cbe and from 1.53 to 1.60 Å when comparing chain *E* of Δ1L2M CXCL13 with those found in PDB entries 5cba and 5cbe. In contrast, the r.m.s.d. values of core domains between any of the combinations of monomers from PDB entries 5cba and 5cbe ranged from 0.55 to 1.13 Å, suggesting that the positioning of helix α1 contributes to the large r.m.s.d. values between the CXCL13 structures in this study and those found for CXCL13 in the complexes with PDB codes 5cba and 5cbe (Supplementary Fig. S4).

Finally, we examined the differences between the N-terminal positions of CXCL13 in this study compared with those from PDB entries 5cba and 5cbe. (We opted to forego analysis of the C-terminal positions, as the monomer with the longest C-terminal density in PDB entries 5cba and 5cbe only extends to Arg72.) While Met CXCL13 and Δ1L2M CXCL13 exhibited densities corresponding to the N-terminus of each construct (*i.e.* Met0 for Met CXCL13 and Met2 for Δ1L2M CXCL13), we observed that the N-terminal positions of CXCL13 in the scFv complexes were not as well resolved. Specifically, the monomers in PDB entry 5cbe exhibited densities beginning at residues Tyr5 and Thr7, while the monomers in PDB entry 5cba exhibited densities beginning at residues Ser8 and Arg10. Despite this, we were able to observe that the N-termini of the CXCL13 monomers from each complex are in intermediate positions between the N-termini of those seen in the Met and Δ1L2M CXCL13 structures, demonstrating the dynamics of the CXCL13 N-terminus prior to the C-*X*-C motif (Fig. 6 ▸ *c*). Similar to our analysis of the high mobility of the C-terminal extension of CXCL13 being captured in overlaid static crystal structures (Figs. 4 ▸ *c* and 5 ▸ *d*), we note that here also such a phenomenon is evident.

### 3.7. Comparisons with murine CXCL13 NMR structures   

**Figure 7:**
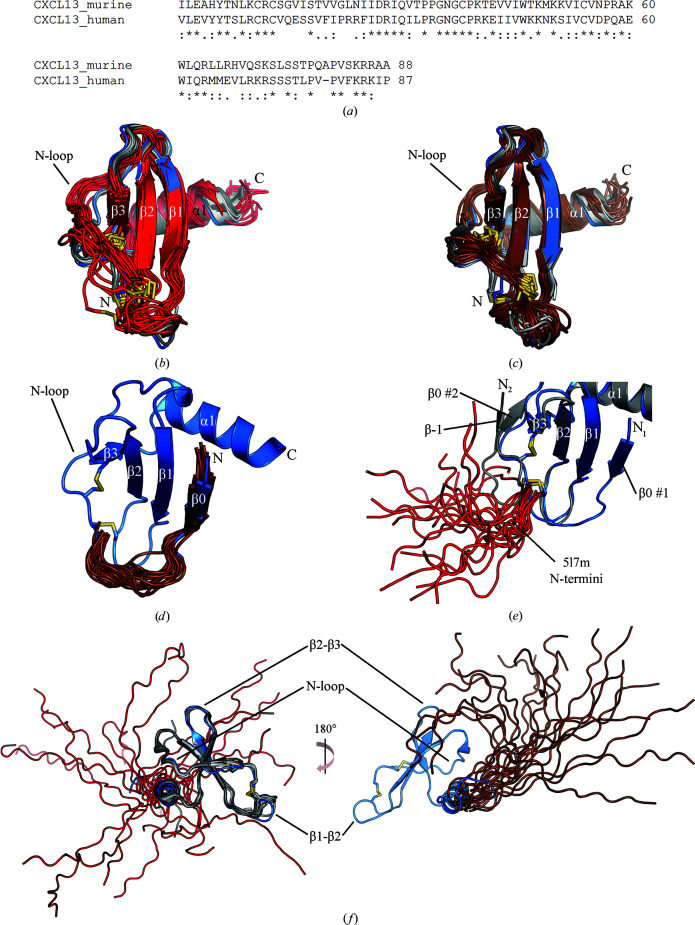
Comparisons between the Met and Δ1L2M CXCL13 crystal structures and the structures of murine CXCL13 found in PDB entries 5l7m and 5izb. Structural features are labeled. (*a*) Alignment of human and murine CXCL13. Asterisks denote amino-acid identity, whereas periods and colons indicate low and high similarity, respectively. (*b*) Alignment of the core domains of the Met and Δ1L2M CXCL13 structures (blue and shades of gray, respectively) with that of the ensemble of WT mCXCL13 atomic positions (red). (*c*) Alignment of the core domain of Met CXCL13 (blue) with that of the ensemble of Met mCXCL13 atomic positions (brown). (*d*) Alignment of the Met CXCL13 structure (blue) with that of the N-terminal atomic positions of Met mCXCL13 (brown). (*e*) Alignment of the Met CXCL13 structure (blue) with that of chain *E* of the Δ1L2M CXCL13 structure (gray) and the N-terminal atomic positions of WT mCXCL13 (red). (*f*) Left: alignment of the Met and Δ1L2M CXCL13 structures minus their N-termini (blue and shades of gray, respectively) with the atomic positions of the C-terminal extension of WT mCXCL13 (red). Right: alignment of the Met CXCL13 structure minus its N-terminus (blue) with the atomic positions of the C-terminal extension of Met mCXCL13 (brown).

In addition to the structures of human CXCL13 in complex with scFvs described above, there also exist two structures of murine CXCL13 (mCXCL13) that have been solved by NMR (PDB entries 5l7m and 5izb; Monneau *et al.*, 2017 ▸). To compare the differences between these structures and our own, we first performed an alignment of the primary sequences of both the human and murine variants using *Clustal Omega* (Madeira *et al.*, 2019 ▸). The alignment shows that the number of residues that are identical in the sequences is only 38 out of 88 possible residues (43%; the murine sequence is one residue longer than the human sequence), although 23 additional residues have a high degree of similarity between the orthologs and a final ten residues are minimally similar (Fig. 7 ▸ *a*). This modest degree of identity between the human and murine orthologs is not uncommon for other chemokines in the C-*X*-C family, although it is at the lower end of the spectrum. For example, the CXCL12 orthologs exhibit 92% amino-acid identity, while the CXCL14 orthologs differ by only two residues (Döring *et al.*, 2014 ▸; Lu *et al.*, 2016 ▸); in contrast, the CXCL11 and CXCL7 orthologs exhibit 68% and ∼50% identity, respectively (Meyer *et al.*, 2001 ▸; Bdeir *et al.*, 2017 ▸). Owing to the differences in the N-terminal sequences of the CXCL13 orthologs (Fig. 7 ▸ *a*), as well as the high degree of dissimilarity in the human and murine CXCR5 N-terminal sequences (data not shown), another region that is important for chemokine–receptor interactions (Allen *et al.*, 2007 ▸), we hypothesize that human and murine CXCL13 may not cross-react with CXCR5 from the other species, although this notion is outside the scope of this work.

The two solution structures of mCXCL13 include the mature WT protein (PDB entry 5l7m) as well as a structure containing an additional initiating methionine at position 0 (PDB entry 5izb), similar to the Met CXCL13 construct described here (for the purposes of discussion, we will call this latter structure Met mCXCL13). By aligning the core domains of the eight monomers from the crystal structures of Met and Δ1L2M CXCL13 with the ensemble of atomic coordinates from the WT mCXCL13 structure (20 structures in total), we observed many similarities along with some striking differences. Specifically, while the β-strands and the C-terminal α1 helix are in largely similar positions, we noticed that the first cysteine in the WT mCXCL13 C-*X*-C motif itself tended to be much closer to the β1 strand compared with the human core domains. We also observed that the N-loop of WT mCXCL13 exhibited a different backbone trajectory when compared with that of the human structures (Fig. 7 ▸ *b*). To our surprise, the N-loop of the Met mCXCL13 structure followed a very similar backbone trajectory to the human structures, although the first cysteine in the C-*X*-C motif still retained its closer proximity to the β1 strand (Fig. 7 ▸ *c*). Compared with the WT mCXCL13 NMR ensemble, the Met mCXCL13 ensemble was also more constrained in its atomic positions (Figs. 7 ▸ *b* and 7 ▸ *c*). These observations suggest that the presence of Met0 in mCXCL13 helps to stabilize the core domain into a conformation that apart from the positioning of the C-*X*-C motif largely mirrors that of the human CXCL13 crystal structures.

We next sought to understand how the presence of Met0 in the murine structure exerted its effect on the core domain. Alignment of the N-terminal atomic positions of Met mCXCL13 with those of Met CXCL13 revealed that the former also exhibits the unique β0 strand present in Met CXCL13 (Figs. 2 ▸ *a* and 7 ▸ *d*). It therefore seems that formation of the β0 strand provides the stabilizing force necessary for movement of the mCXCL13 N-loop into a position that reflects the human CXCL13 structures. Indeed, alignment of the N-terminal atomic positions of WT mCXCL13 with those of Met CXCL13 and chain *E* of Δ1L2M CXCL13 shows that the N-terminus of WT mCXCL13 is highly mobile, sampling multiple positions in space that are intermediate to the β0 and β−1 strands formed by Met and Δ1L2M CXCL13, respectively (Fig. 7 ▸ *e*). These N-terminal positions of mCXCL13 are reminiscent of the N-terminal positions of human CXCL13 in complex with scFvs described earlier (Figs. 6 ▸ *c* and 7 ▸ *e*).

Finally, we examined the positions of the C-terminal extension in mCXCL13 and compared them with those seen in the Met and Δ1L2M CXCL13 structures (Fig. 7 ▸ *f*). Alignment of the atomic coordinates of the C-terminal extension of eight monomers from the Met and Δ1L2M CXCL13 structures with those of the WT mCXCL13 demonstrated that WT mCXCL13 also exhibits a striking degree of mobility in its C-terminal extension, sampling a space that covers a full 360° perpendicular to the α1 helical axis (Fig. 7 ▸ *f*, left). We note that these positions expand upon the distinctive trajectories of the C-terminal extension observed in the human CXCL13 structures (Fig. 5 ▸ *d*). Remarkably, alignment of the Met CXCL13 structure with the atomic coordinates of the C-terminal extension of Met mCXCL13 revealed that the C-terminal extension of Met mCXCL13 is much more restricted in three-dimensional space, sampling trajectories that are largely directed away from the core domain, somewhat analogous to the trajectory seen in the Met CXCL13 crystal structure (Fig. 7 ▸ *f*, right). Similar to our earlier suggestion that the N-loop changes position when the β0 strand is present, we propose that the presence of the β0 strand in Met mCXCL13 provides a stabilizing force that helps to restrict the positioning of the C-terminal extension (relative to that in WT mCXCL13), although we note that steric effects may also play a role.

## 4. Conclusions   

CXCL13 binds to a sole receptor, CXCR5, to elicit its chemotactic activity (Moschovakis *et al.*, 2017 ▸). This activity is important for adaptive immune responses by regulating B-cell and TFH-cell localization within lymph nodes and other secondary lymphoid organs. Indeed, mice with a whole-body CXCR5 knockout display defects in secondary lymphoid-tissue architecture (Förster *et al.*, 1996 ▸). As our understanding of the CXCL13–CXCR5 axis continues to grow, it is becoming clearer that this interacting pair may represent a valuable drug target in multiple disease conditions, including autoimmune disorders and cancers (Airoldi *et al.*, 2008 ▸; Biswas *et al.*, 2014 ▸; Bürkle *et al.*, 2007 ▸; Charbonneau *et al.*, 2013 ▸; El-Haibi *et al.*, 2011 ▸, 2013 ▸; Singh *et al.*, 2009 ▸, 2014 ▸; Dupuis *et al.*, 2006 ▸; Bao *et al.*, 2020 ▸; Klimatcheva *et al.*, 2015 ▸). Despite this, no drug candidates have successfully been developed to target the receptor, nor are there any structural data on the receptor to aid in their development. For these reasons, we opted to perform an initial characterization of the ligand CXCL13 in order to better understand how its N-terminal length and side-chain composition affect the activity of CXCR5. In addition, we utilized two of these constructs to solve the first structures of uncomplexed human CXCL13. We hope that these structures will aid researchers who wish to perturb the signaling axis via targeting of CXCL13 or to perform *in silico* approaches aimed at understanding CXCL13–CXCR5 interactions.

We generated three N-terminal mutants of CXCL13 to study their behavior on CXCR5-expressing cells, namely Met, V1M and Δ1L2M CXCL13 (Table 2 ▸). Our original interest was in investigating the activity of Met CXCL13, as the presence of an initiating methionine in other chemokines has been shown to result in varying effects. For example, Met CXCL12 is known to function as an agonist of CXCR4, whereas Met CCL5 is a potent antagonist of CCR5 (Proudfoot *et al.*, 1996 ▸; Rosenberg *et al.*, 2019 ▸). The exact mechanisms by which the extra methionine residue affects the receptor activation state is likely to vary on a case-by-case basis, but we speculate that it is likely to be rooted in the necessary conformational changes in the orthosteric pocket of the receptor to accommodate the extra methionine residue or in whether such a conformational change is even possible for every given receptor. In the case of CXCR4, which can accommodate the Met CXCL12 N-terminus, the orthosteric pocket probably has similar conformations induced by either WT CXCL12 or Met CXCL12 to trigger activation. In the case of Met CCL5, the extra methionine may prevent the N-terminus of CCL5 from entering the orthosteric site of CCR5 entirely, in which case the ligand would be interacting with the receptor primarily through receptor N-terminus and chemokine core domain interactions. Indeed, it has been suggested that C-C chemokines attain most of their receptor-binding affinity through residues in their core domains, whereas C-*X*-C chemokines rely on their N-termini for high affinity for their cognate receptors (Qin *et al.*, 2015 ▸; Hanes *et al.*, 2015 ▸). Assuming instead that Met CCL5 can insert its N-terminus into the CCR5 orthosteric site, the conformational change involved presumably hampers other CCL5–CCR5 interactions necessary for receptor activation or the ability of the receptor to adopt the active state altogether, resulting in the observed antagonistic effect. The functional assay used in this study (calcium flux) allowed us to observe that Met CXCL13 functions as an agonist of CXCR5 (Fig. 1 ▸). This indicates that the conformation of the orthosteric pocket induced by CXCL13 with a Met0 is still capable of forming appropriate contacts with other CXCL13 residues to induce the active conformation of CXCR5.

We also studied the activities of V1M and Δ1L2M CXCL13, mutants that were designed using a ‘methionine scanning’ approach in order to compare their activities with those of WT and Met CXCL13 (Table 2 ▸). By moving the methionine, we observed that both of these additional constructs were still able to induce CXCR5 activation (Fig. 1 ▸). Analysis of the potencies of these constructs allowed us to suggest that the length of the CXCL13 N-terminus is fine-tuned to be most active at its native length, even with a substitution of Val1 by the much bulkier Met1 in V1M CXCL13. The insertion mutant Met CXCL13 suffers a minor penalty in receptor activation, which is likely to be owing to steric constraints imposed by the orthosteric pocket of CXCR5. The lower activity of Δ1L2M CXCL13 could arise either from diminished binding to CXCR5 or a decreased ability to trigger a receptor conformational change to the active state. Although we did not perform binding studies in this paper, we believe that the loss of binding affinity was a contributing factor since other C-*X*-C chemokines obtain binding affinity through their N-termini (Qin *et al.*, 2015 ▸; Hanes *et al.*, 2015 ▸). Since the methionine at position 2 would still be able to form hydrophobic interactions similar to the native Leu2, we believe that the Δ1L2M mutant is likely to be able to form similar contacts to those of WT CXCL13 other than those lost by removing residue 1. The loss of contacts at residue 1 are a likely culprit for the significantly decreased efficacy of this construct, suggesting that residue 1 plays a critical role in triggering the activation of CXCR5. Altogether, our functional data demonstrate that CXCR5 is capable of tolerating minor length and side-chain variations in the extreme N-terminal residues of its ligand CXCL13, especially when these variations involve increased bulk in the orthosteric site rather than a loss of contacts. Indeed, we speculate that a loss of natural contacts may represent a major contributing factor explaining why modifications to the N-termini of chemokines frequently result in antagonistic variants (Allen *et al.*, 2007 ▸; Fernandez & Lolis, 2002 ▸), whereas the mutants in this work with most contacts retained all function as partial agonists.

We reasoned that structural information would allow rational targeting of the ligand (Smith *et al.*, 2014 ▸) and would be of use in generating an *in silico* model of the CXCL13–CXCR5 complex. Although we attempted to crystallize all of the CXCL13 constructs expressed and purified in this study, we were unable to crystallize WT CXCL13 and the V1M variant. While the core domain of CXCL13 exhibited a canonical chemokine fold in Met and Δ1L2M CXCL13, we observed striking variations in the N- and C-terminal positions of the ligand. We found that the N-terminus of Met CXCL13 was not disordered as in other chemokine structures, but instead formed an extra parallel β-strand interaction (β0) with β1 in the core domain (Figs. 2 ▸ *a* and 2 ▸ *b*), seemingly mediated by the initiating methionine itself (Fig. 3 ▸). Such an observation has also been found to occur in a murine variant of Met CXCL13 (Fig. 7 ▸ *d*). In contrast, the Δ1L2M CXCL13 structure had a completely different orientation of the N-terminus, in which it turned away from the core domain (Fig. 4 ▸ *b*). Comparisons of these radically different N-terminal positions demonstrate the extreme flexibility of the N-terminus (Figs. 5 ▸ *b* and 5 ▸ *c*). Since both the Met and Δ1L2M variants functioned as agonists (Fig. 1 ▸), we can deduce that in both cases the N-terminus of CXCL13 must be able to dissociate from these β-strand interactions in order to insert into the orthosteric site of CXCR5 and induce receptor activation. Indeed, it has previously been shown that the β0 strand in the Met mCXCL13 NMR structure is in slow exchange with a flexible state, supporting this notion (Monneau *et al.*, 2017 ▸). It is possible that the β-strand interactions found within these structures may simply be artifacts of the crystallization process and/or be mutation-specific. In addition, we compared these N-terminal positions with those of human CXCL13 bound to scFv molecules (Tu *et al.*, 2016 ▸) and a solution structure of WT mCXCL13 (Monneau *et al.*, 2017 ▸). These comparisons (Figs. 6 ▸ *c* and 7 ▸ *e*) demonstrate that the N-terminus of CXCL13 is highly dynamic and is capable of sampling three-dimensional space intermediate to the positions found in the crystal structures contained here. Taken together, these structures support the idea that the N-terminus of CXCL13 exhibits striking flexibility owing to its inherent disorder, which is necessary for its interaction with the orthosteric site of the receptor (Allen *et al.*, 2007 ▸; Fernandez & Lolis, 2002 ▸).

In addition to the observed N-terminal flexibility, our structures also allowed us to visualize the high flexibility of the CXCL13 C-terminal extension. In particular, alignment of the seven monomers within the Δ1L2M CXCL13 structure as well as the Met CXCL13 monomer highlighted that they varied quite considerably in the positioning of their C-terminal extensions (Figs. 4 ▸ *c* and 5 ▸ *d*). This was further demonstrated by comparisons with the murine CXCL13 solution structures, which showed remarkable flexibility that was partially restricted in the Met mCXCL13 structure (Fig. 7 ▸ *f*). Considering that these eight aligned monomers from the Met and Δ1L2M CXCL13 structures, excluding their N-termini, are very similar up to residue 72 (Fig. 5 ▸ *a*), this indicates that at least the last 15 residues in human CXCL13 (Ser73–Pro87) are intrinsically disordered. Such a feature has been observed in a subset of other chemokines. In particular, C-terminal extensions have been observed to occur in the chemokines XCL1, CXCL9, CXCL12γ, CCL16, CCL21, CCL25 and CCL28, and have been implicated in both glycosaminoglycan (GAG) binding on the surface of cells and, in some cases, antimicrobial activity (Moussouras *et al.*, 2020 ▸). Indeed, the C-terminal extension of mCXCL13 has previously been linked to GAG binding (Monneau *et al.*, 2017 ▸). It is likely that the C-terminal extension of human CXCL13 contributes to GAG binding, although this remains to be determined by experimental studies.

While the N- and C-termini of human CXCL13 appear to be intrinsically disordered, we observed that the core domain of CXCL13 is quite rigid (Fig. 5 ▸ *a*). We also compared the core domain from Met CXCL13 and that from the Δ1L2M CXCL13 structure with those from the four monomers seen in CXCL13–scFv complexes in PDB entries 5cba and 5cbe, finding that the largest change was in the angling of the α1 helix with respect to the β-sheet (Figs. 6 ▸ *a* and 6 ▸ *b*). This change seems to be caused by interactions with the scFv molecules and symmetry mates in these structures, indicating that it is likely to be induced rather than a feature of the core domain itself. We also observed that the core domain of mCXCL13 largely matches those seen in human CXCL13, with Met mCXCL13 looking more similar than WT mCXCL13 (Figs. 7 ▸ *b* and 7 ▸ *c*). Collectively, these observations support the idea that the core domain of CXCL13 is quite rigid, which is in stark contrast to its extreme N- and C-terminal flexibility.

An interesting feature of our structures is that it is not possible to observe a canonical C-*X*-C chemokine dimer in either of them. C-*X*-C chemokines are known to dimerize via antiparallel β-strand interactions mediated by the β1 strand in each monomer, leading to an overall six-stranded β-sheet (Allen *et al.*, 2007 ▸; Miller & Mayo, 2017 ▸; for a representative example, see Fig. 2 ▸ *d*). Chemokines engage with GAGs in their dimeric forms in order to regulate local concentrations of ligand in order to establish the gradients necessary for chemotaxis (Handel *et al.*, 2005 ▸; Verkaar *et al.*, 2014 ▸), prompting the notion that GAGs may induce a six-stranded WT CXCL13 dimer. Other questions arise when considering whether the crystallographic Met CXCL13 dimer (Fig. 2 ▸ *c*) or an aggregate such as that seen in the Δ1L2M CXCL13 structure (Fig. 4 ▸ *a*) are only mutational and/or crystallographic artifacts or instead are stable complexes that could exist in solution. If the latter case was true, these would represent entirely new modes of chemokine oligomerization, with possible implications for GAG binding.

We note that in all structures of human CXCL13 (Met CXCL13, Δ1L2M CXCL13 and the scFv complexes from PDB entries 5cba and 5cbe) it is observed that the β1 strand interacts with additional β-strands. In the Met CXCL13 structure this is represented by the β0 strand, whereas in the Δ1L2M CXCL13 structure the interacting partner is instead the β−1 strand from an adjacent monomer (Figs. 2 ▸ *a* and 4 ▸ *b*, respectively). Examination of the structures with PDB codes 5cba and 5cbe shows that in each structure the β1 strand of CXCL13 engages in an antiparallel β-strand interaction with a β-strand localized in a loop within the variable region of the heavy chain of the scFv. The authors note that this interaction seems to be mediated exclusively through backbone inter­actions between the β-strands and is not stabilized by any amino-acid side chains (Tu *et al.*, 2016 ▸). While we describe how Met0 stabilizes the formation of the β0 strand via intermolecular and intramolecular interactions (Fig. 3 ▸), we do not observe Met2 in the Δ1L2M structure to play a similar role. Instead, we observe that the interaction between β1 in one monomer and β−1 in an adjacent monomer is limited to backbone interactions (data not shown), and that an induced β0 strand in the N-loop helps to stabilize the positioning of β−1 (Fig. 4 ▸ *b*). Taken together, it seems to us that stabilization of the β1 strand with an interacting partner is required for the purposes of crystallization. As we were unable to crystallize either WT or V1M CXCL13, we wonder whether similar stabilizing interactions with β1 could not easily occur between monomers with an N-terminus of native length. This notion suggests that the β1 strands of CXCL13 monomers may not have an intrinsically high affinity for one another. Moreover, stabilization of β1 alone may not be sufficient to help stabilize the constructs for crystallization, as in our structures and those seen in PDB entries 5cba and 5cbe there are additional contacts other than those mediated just by β1 backbone interactions that presumably help to lower the energy of the system.

To summarize, we have provided insights into the functionality of the N-terminus of CXCL13 and the degree to which minor variability can be tolerated by CXCR5 for activation. Furthermore, we have solved the first two uncomplexed crystal structures of human CXCL13. Our structures show high degrees of flexibility in the N-terminus of the ligand as well as the C-terminal extension, and support the concept that the core domain is fairly rigid. Our structures also pave the way for studies examining the binding of CXCL13 to GAGs as well as its dimerization characteristics. We envision that the information generated from our studies will aid in efforts to better understand how the CXCL13–CXCR5 signaling axis functions on a molecular level, as well as how it can be perturbed, for both basic science and therapeutic benefit in the future.

## Supplementary Material

PDB reference: CXCL13, 7jny

PDB reference: Δ1L2M variant, 6vgj

Supplementary Table and Figures. DOI: 10.1107/S2059798320011687/nj5292sup1.pdf

## Figures and Tables

**Table 1:** Crystallographic statistics for the Met and Δ1L2M CXCL13 crystal structures Each data set was obtained from a single crystal, and the resulting data-collection and refinement statistics are shown. Values in parentheses are for the highest resolution shell.

	Met CXCL13 (PDB entry 7jny)	Δ1L2M CXCL13 (PDB entry 6vgj)
Data collection		
Space group	*P*3_1_21	*P*12_1_1
*a*, *b*, *c* (Å)	48.97, 48.97, 80.28	69.76, 41.57, 111.77
α, β, γ (°)	90.0, 90.0, 120.0	90.0, 102.1, 90.0
Resolution (Å)	50.00–1.88 (1.91–1.88)	50.00–2.52 (2.56–2.52)
*R*_p.i.m._	0.027 (0.448)	0.058 (0.919)
CC_1/2_	1.003 (0.754)	0.990 (0.356)
〈*I*/σ(*I*)〉	20.12 (1.58)	12.39 (0.94)
Completeness (%)	99.4 (94.5)	98.6 (86.5)
Multiplicity	4.8 (2.9)	4.8 (2.6)
Refinement		
Resolution (Å)	42.41–1.88	47.82–2.52
No. of reflections	9388	21421
*R*_work_/*R*_free_	0.19/0.22	0.25/0.28
No. of atoms		
Protein	661	4220
Water	75	29
*B* factors (Å^2^)		
Protein		
Overall	35.5	55.2
Chain *A*		54.7
Chain *B*		50.9
Chain *C*		51.3
Chain *D*		56.5
Chain *E*		56.2
Chain *F*		55.3
Chain *G*		59.7
Water	37.8	31.1
R.m.s deviations		
Bond lengths (Å)	0.010	0.011
Bond angles (°)	1.290	1.080

**Table 2:** CXCL13 constructs discussed in this paper The N-terminal sequences of the proteins up to the C-*X*-C motif (in red) are shown. Both EC_50_ and efficacy values (obtained from calcium-flux experiments; Fig. 1 ▸) are also listed for each construct. Numbers in parentheses indicate 95% confidence intervals. Residues up to position 9 are numbered in the bottom row for clarity.

Construct	N-terminal sequence	EC_50_ (n*M*)	Efficacy (%)
WT CXCL13	VLEVYYTSLRCRC…	2.49 (1.97, 3.12)	100
Met CXCL13	MVLEVYYTSLRCRC…	26.3 (21.8, 31.7)	86.8
V1M CXCL13	MLEVYYTSLRCRC…	5.64 (4.36, 7.26)	93.6
Δ1L2M CXCL13	MEVYYTSLRCRC…	133 (92.0, 194)	55.1
	0123456789----…		
