# Supplementary material for: The N-terminal length and side-chain composition of CXCL13 affect crystallization, structure and functional activity
Source: Acta Crystallogr D Struct Biol. 2020 Sep 25;76(Pt 10):1033–49. doi: 10.1107/S2059798320011687 (PMC7543660; doi:10.1107/S2059798320011687)
Supplement: Supplementary file 1 [file d-76-01033-sup1.pdf]

STRUCTURAL BIOLOGY

Volume 76 (2020)

Supporting information for article:

CXCL13 N-terminal length and side-chain composition affect crystallization, structure and functional activity

Eric M. Rosenberg, James Herrington, Deepa Rajasekaran, James W. Murphy, Georgios Pantouris and Elias J. Lolis

Table S1 Oligonucleotide sequences that were used as primers during mutagenesis reactions to generate the CXCL13 mutants discussed in the text.

Note that EK-CXCL13 produces WT CXCL13 after cleavage of the N-terminus

Construct	Oligonucleotide sequence
V1M	F: 5'- GAAGGAGATATACATATGCTGGAAGTGTATTATACC -3'<br>R: 5'- GGTATAATACACTTCCAGCATATGTATATCTCCTTC -3'
Δ1L2M	F: 5'- GAAGGAGATATACATATGGAAGTGTATTATACCAGC -3'<br>R: 5'- GCTGGTATAATACACTTCCATATGTATATCTCCTTC -3'
EK-CXCL13 (WT)	F: 5'- GGAGATATACATATGGATGATGATGATAAAGTGCTGGAAGTG -3'<br>R: 5'- CACTTCCAGCACTTTATCATCATCATCCATATGTATATCTCC -3'

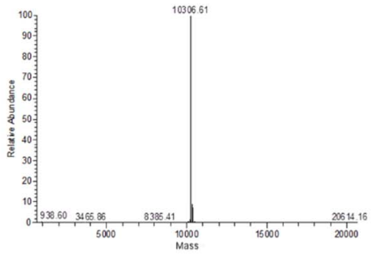

(*a*)

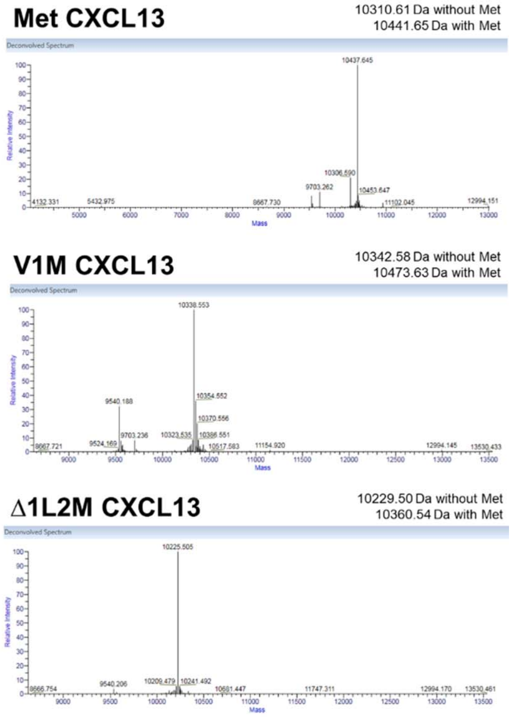

(*b*)

Figure S1 LC-MS profiles of the purified CXCL13 constructs used in this paper. The mass spectrometry profiles are displaying the monoisotopic molecular weights (rather than the average). (*a*) LC-MS profile of recombinantly purified WT CXCL13. The expected monoisotopic mass for WT CXCL13 is 10,310.61 Da; the largest peak differs by 4 Da, which can be attributed to oxidation of the four cysteines to form two disulfide bonds. (*b*) LC-MS profiles of the other CXCL13 constructs discussed in the text. For each construct, the expected monoisotopic mass both with and without the initiating methionine residue is listed. The largest peaks differ from the expected monoisotopic masses differ by approximately 4 Da, which can be attributed to oxidation of the four cysteines in each construct to form two disulfide bonds. Note that Met CXCL13 seems to be a mixed species, with a small peak corresponding to the construct lacking the initiating methionine (yielding WT CXCL13). V1M CXCL13 exhibits one large peak at ~9540 Da, which is either a contaminant or a degradation product**.**

RMSDs, Cα (Å)								
Structure and chain ID		Δ1L2M						
		A	B	C	D	E	F	G
Δ1L2M	A	-	0.38	0.50	0.43	0.58	0.94	0.42
	B	0.38	-	0.52	0.44	0.65	0.85	0.34
	C	0.50	0.52	-	0.44	0.68	0.92	0.62
	D	0.43	0.44	0.44	-	0.66	0.82	0.51
	E	0.58	0.65	0.68	0.66	-	0.82	0.66
	F	0.94	0.85	0.92	0.82	0.82	-	0.96
	G	0.42	0.34	0.62	0.51	0.66	0.96	-

RMSDs, Backbones (Å)								
Structure and chain ID		Δ1L2M						
		A	B	C	D	E	F	G
Δ1L2M	A	-	0.42	0.50	0.42	0.59	0.94	0.46
	B	0.42	-	0.61	0.52	0.70	0.86	0.43
	C	0.50	0.61	-	0.45	0.69	0.91	0.68
	D	0.42	0.52	0.45	-	0.66	0.82	0.60
	E	0.59	0.70	0.69	0.66	-	0.81	0.70
	F	0.94	0.86	0.91	0.82	0.81	-	0.99
	G	0.46	0.43	0.68	0.60	0.70	0.99	-

Figure S2 RMSDs of the N-termini and core domains from the Δ1L2M CXCL13 crystal structures. Each chain from the structure was truncated after residue 72 in the mature WT sequence to eliminate their C-terminal extensions. The top table provides the RMSD values for Cα atoms within the chains, while the bottom table provides the values for the entire backbone of each chain.

RMSDs, Cα (Å)

Structure and chain ID		Met	Δ1L2M A	Δ1L2M B	Δ1L2M C	Δ1L2M D	Δ1L2M E	Δ1L2M F	Δ1L2M G
Met		-	0.57	0.98	1.05	0.86	0.88	1.03	0.94
Δ1L2M	A	0.57	-	0.35	0.47	0.32	0.56	0.85	0.38
	B	0.98	0.35	-	0.52	0.41	0.67	0.78	0.33
	C	1.05	0.47	0.52	-	0.40	0.67	0.88	0.61
	D	0.86	0.32	0.41	0.40	-	0.67	0.83	0.47
	E	0.88	0.56	0.67	0.67	0.67	-	0.75	0.68
	F	1.03	0.85	0.78	0.88	0.83	0.75	-	0.88
	G	0.94	0.38	0.33	0.61	0.47	0.68	0.88	-

RMSDs, Backbones (Å)

Structure and chain ID		Met	Δ1L2M A	Δ1L2M B	Δ1L2M C	Δ1L2M D	Δ1L2M E	Δ1L2M F	Δ1L2M G
Met		-	0.61	1.02	1.07	0.87	0.89	1.05	0.97
Δ1L2M	A	0.61	-	0.40	0.48	0.32	0.56	0.86	0.39
	B	1.02	0.40	-	0.63	0.51	0.72	0.80	0.37
	C	1.07	0.48	0.63	-	0.41	0.68	0.87	0.67
	D	0.87	0.32	0.51	0.41	-	0.67	0.83	0.53
	E	0.89	0.56	0.72	0.68	0.67	-	0.74	0.68
	F	1.05	0.86	0.80	0.87	0.83	0.74	-	0.87
	G	0.97	0.39	0.37	0.67	0.53	0.68	0.87	-

Figure S3 RMSDs of core domains from the Met and Δ1L2M CXCL13 crystal structures. Each chain from the two crystal structures was truncated to contain only the core domain (i.e., beginning at the C-X-C motif [residue 11] and ending at residue 72 in the mature WT sequence). The top table provides the RMSD values for Cα atoms within the chains, while the bottom table provides the values for the entire backbone of each chain.

RMSDs, Cα (Å)							
Structure and chain ID		Met	Δ1L2M	5CBA		5CBE	
			E	E	F	E	F
Met		-	0.84	1.38	1.37	1.31	1.23
Δ1L2M	E	0.84	-	1.47	1.49	1.55	1.55
5CBA	E	1.38	1.47	-	0.52	0.95	1.11
	F	1.37	1.49	0.52	-	0.87	1.00
5CBE	E	1.31	1.55	0.95	0.87	-	1.03
	F	1.23	1.55	1.11	1.00	1.03	-

RMSDs, Backbones (Å)							
Structure and chain ID		Met	Δ1L2M	5CBA		5CBE	
			E	E	F	E	F
Met		-	0.85	1.42	1.44	1.36	1.25
Δ1L2M	E	0.85	-	1.53	1.55	1.60	1.56
5CBA	E	1.42	1.53	-	0.55	0.89	1.13
	F	1.44	1.55	0.55	-	0.86	1.08
5CBE	E	1.36	1.60	0.89	0.86	-	1.06
	F	1.25	1.56	1.13	1.08	1.06	-

Figure S4 RMSDs of core domains from monomers in the Met, Δ1L2M 5CBA, and 5CBE structures. Each chain from the two crystal structures was truncated to contain only the core domain (i.e., beginning at the C-X-C motif [residue 11] and ending at residue 68 in the mature WT sequence). The top table provides the RMSD values for Cα atoms within the chains, while the bottom table provides the values for the entire backbone of each chain. Note that, for brevity, only chain E from the Δ1L2M structure was used in calculations.
